# Sharing the Space With the “Victim” Can Increase Help Rates. A Study With Virtual Reality

**DOI:** 10.3389/fpsyg.2021.729077

**Published:** 2021-09-08

**Authors:** Anna Spagnolli, Mariavittoria Masotina, Mattia Furlan, Patrik Pluchino, Massimiliano Martinelli, Luciano Gamberini

**Affiliations:** ^1^Department of General Psychology, University of Padova, Padua, Italy; ^2^Human Inspired Technologies Research Centre, University of Padova, Padua, Italy

**Keywords:** helping behavior, spatial arrangement, emergency, fire, virtual reality, social categorization

## Abstract

A typical protocol for the psychological study of helping behavior features two core roles: a help seeker suffering from some personal or situational emergency (often called “victim”) and a potential helper. The setting of these studies is such that the victim and the helper often share the same space. We wondered whether this spatial arrangement might affect the help rate. Thus, we designed a simple study with virtual reality in which space sharing could be manipulated. The participant plays the role of a potential helper; the victim is a humanoid located inside the virtual building. When the request for help is issued, the participant can be either in the same spatial region as the victim (the virtual building) or outside it. The effect of space was tested in two kinds of emergencies: a mere request for help and a request for help during a fire. The analysis shows that, in both kinds of emergencies, the participants were more likely to help the victim when sharing the space with it. This study suggests controlling the spatial arrangement when investigating helping behavior. It also illustrates the expediency of virtual reality to further investigate the role of space on pro-social behavior during emergencies.

## Introduction

The study of helping behavior has attracted the scholars’ interest since the early works of Darley and Latanè in the 60s (Darley and Latanè, 1968) and has been recently transposed to virtual reality (VR; e.g., Slater et al., 2013). Indeed, classic phenomena such as the bystander effect (Kozlov and Johansen, 2010) or outgroup discrimination (Slater et al., 2013) in helping behavior have been successfully replicated in VR.

The reasons for using VR when studying the response to a help request are manifold. First, since information about facts or states might be inaccessible through introspection (Nosek et al., 2011) or affected by memory biases (Hyman and Loftus, 1998), self-reporting is increasingly replaced or complemented by behavioral measures (e.g., Monaro et al., 2018; D’Errico et al., 2020; Papapicco et al., 2021). VR allows the accurate recording of behavioral measures for subsequent inspection and analysis (Pan and Hamilton, 2018). Second, VR provides a perceptually vivid and responsive setting when a study *in situ* is not safe; indeed, these qualities motivate its use not only in the study of emergency behavior but also in its training (e.g., the commercial FLAIM Trainer^TM^^1^ or the TEP platform by the United States navy^2^). Finally, a virtual environment can implement the experimental design of a study in a controlled yet affordable way, hardly achievable with a physical setting (Blascovich et al., 2002).

The studies carried out so far with or without VR have shown that situational, socio-cultural, and personal factors affect the decision to comply with a help request. Situational factors include concurrent tasks and time pressure (Darley and Batson, 1973), the presence of other potential helpers (Darley and Latanè, 1968), and the ambiguity and the seriousness of the emergency (Schwartz and Clausen, 1970; Clark and Word, 1974; Fischer et al., 2006; Lovreglio et al., 2015). Socio-cultural factors include the ethnicity of the victim (Dasgupta, 2004), the gender of the potential helper (Senneker and Hendrick, 1983), the gender of the bystander (Schwartz and Clausen, 1970), and the anonymity of the potential helper (Schwartz and Gottlieb, 1980).

One aspect that is usually not controlled in the literature on helping behavior is the relative spatial position of the potential helper and the help seeker (henceforth, the victim). We have examined the procedure of classic studies of helping behavior as well as the more recent studies using virtual reality; we found that the victim needing help and the potential helper are often in the same, delimited space and mutual sight (Darley and Latanè, 1968; Darley and Batson, 1973; Darley et al., 1973; Clark and Word, 1974; Gaertner, 1975; Gaertner and Dovidio, 1977; Senneker and Hendrick, 1983; Harari et al., 1985; Shotland and Heinold, 1985; Levine et al., 2005; Gillath et al., 2008; Kunstman and Plant, 2008; van den Bos et al., 2009; Slater et al., 2013; Zanon et al., 2014; Gamberini et al., 2015). Sometimes, they even share some danger or collaborate on the same task (Darley and Latanè, 1968; Gaertner, 1975; Gaertner and Dovidio, 1977; Senneker and Hendrick, 1983; Kunstman and Plant, 2008; Zanon et al., 2014).

We wondered whether letting the victim and potential helper share the same space might introduce a confound in the procedure. The reasons are offered by the social categorization framework (SC, Tajfel et al., 1979), showing that people tend to be more pro-social when the person in need is part of their ingroup (e.g., Dasgupta, 2004; Kunstman and Plant, 2008) and that the participants’ mutual position can split them into an ingroup and an outgroup (seatings’ spatial arrangement, Gaertner et al., 1993; neighborhood, Bernardo and Palma-Oliveira, 2016). Therefore, we hypothesized that people might be more pro-social when the victim is in the same space as the helper. The present study explores this hypothesis by manipulating the victim’s inclusion in the potential helper’s space and observing the help rate. Moreover, we wondered whether the effect of space—if any—would persist in front of a more blatant emergency; therefore, we varied the type of emergency, which could be either a vocal request for help or a request for help during a fire. The study follows a between-participant, 2 × 2 design with four experimental conditions (indoor without fire; outdoor without fire; indoor with fire; outdoor with fire). We hypothesized a higher help rate when the participants are indoor (i.e., in the same space as the victim) and that the effect of space persisted regardless of the type of emergency. With this study, we aim to contribute to the research area of pro-social behavior by highlighting a factor that can be worth methodological attention.

## Method

### The Virtual Environment

**FIGURE 1:**
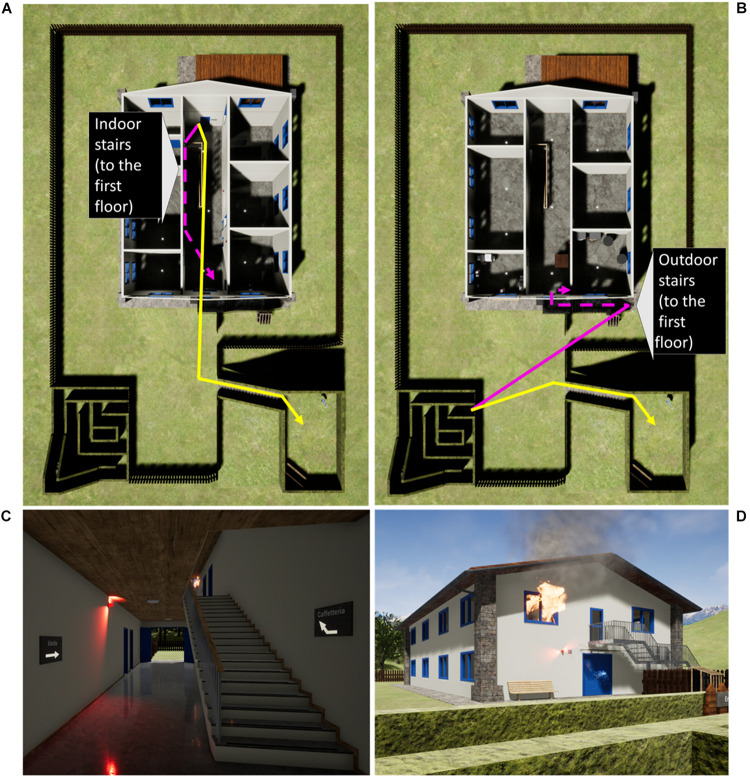
The VE. The bird-eye views of the VE show the indoor **(A)** and outdoor **(B)** bifurcation (yellow line, route to the session’s endpoint; purple, route to the victim), while the first-person views show the fire effects door, **(C)** and outdoor **(D)**.

The study was conducted in a virtual environment (VE). The participant was the potential helper, while a humanoid was the victim seeking help. We placed no other character in the VE to prevent interference due to a bystander effect (Latanè and Darley, 1970). The VE contained one building surrounded by a garden, creating two distinct indoor and outdoor regions. The victim was always indoor, whereas the participant could be either inside the building or outdoors when receiving the request for help, depending on the experimental condition. The participant’s position was manipulated by instructing them to reach a plate in the building or in the garden. Reaching that plate triggered the request for help. We also manipulated the type of emergency: the victim would just issue a verbal help request, or the request would appear right after the burst of a fire in the virtual building. The fire was visible from both inside and outside the building (Figures 1B,C).

To obtain a behavioral measure of compliance with the help request, we instructed the participants to go back to the hilltop after reaching the plate. At this point, the help request was issued, and two possible routes were available: to the endpoint of the session, i.e., the hilltop in the garden, or to the victim’s location, i.e., the cafeteria inside the building (Figures 1A,B). In this way, the route taken by the participant indicated whether they complied or not with the request for help.

In the conditions with a fire, visual and acoustic effects started a few seconds before the request for help: some flames, some free-floating cables emitting electric sparks, a red alarm light, an initial small blast sound, and an intermittent alarm siren. These effects were perceivable from both inside and outside the virtual building (Figures 1C,D).

The VE and the humanoid were developed using Unreal Engine v. 4.18.3^3^, Blender 2.77a, Embarcadero Delphi XE2 Professional, GIMP 2.8.18, MakeHuman 1.1.0, Microsoft Visual Studio Community 2015. Audacity 2.1.2 was used to improve the avatars’ recorded voice, and TocaEdit Controller emulator 3.2.8.77 was used to program the input interface. Pilot tests were conducted, and some improvements to the VE were made consequently; for instance, we made the outdoor staircase invisible in the indoor condition to prevent it from being used. Also, the storage room door position was changed since its initial lateral position made it difficult to take the door.

### Setting

The experiment took place in a laboratory where the VE was projected on a 225 × 300 cm screen, located 290 cm from the standing participant. During the navigation, the light sources were the projected VE (60% screen brightness) and a LED strip on the ceiling halfway between the participant and the screen. The sound was produced by a Dolby surround system, constituted of four speakers located in the room’s upper corners. The participant interacted with the VE *via* a Trust GX 30 controller. Specifically, the stick on the left controlled the movement (right-left and forward-backward), while the stick on the right controlled the view shifts (right-left, up-down). To start or end a session, the participant had to press a triangle button. Further controls were not needed: to open doors or climb stairs in the VE, the participants would just need to approach them. The walking speed was set constantly at 0.30 m/s. A picture of the setting is provided as the Supplementary Material.

### Procedure

Before starting the experiment, the participant was asked to read the informed consent (see section “Ethics” for Ethics in this study). The participants then watched a video meant to reduce any difference in anxiety between them, since this could affect the help rate (von Dawans et al., 2018); the video featured a slow, bright, and peaceful underwater world accompanied by some soft music (as in Piferi et al., 2000). Then they were asked to fill in a questionnaire collecting information about their age, gender, the fulfillment of the inclusion criteria (described in section “Participants”), their expertise with videogames, and their level of state anxiety. Then, the VR viewpoint was adjusted to the participant’s height, and the three interaction sessions with the VE started. At the start of each session, the participants would find themselves on the hilltop in the virtual garden.

#### First Session: Training

This phase’s goal was to allow the participants to practice using the joypad and the movements in the VE. Participants were asked to go through the labyrinth in the VE’s garden at least three times (or more, if they wished). The experimenter remained close to assist them in case of need.

#### Second Session: Exploration

Before starting, the participant was instructed to visit a few selected rooms in the building and was shown the access route to the building stairs, either indoor or outdoor depending on the experimental condition. This phase allowed the participant to get familiar with the route needed in the experimental phase and to notice the humanoid in the cafeteria. When the navigation started, the researcher left the room. The participant’s activity was recorded in the VE’s log to ascertain that the instructions were followed. Any opportunity of engaging with the humanoid was prevented by depicting him busy with a phone conversation (“Hello, this is Luca, how are you? Yes… Good …. Sure”). Upon completing the exploration, the participants were given a blueprint of the VE and asked to write down the rooms’ names, mark the entrance position, and mark the room in which they saw the humanoid. This exercise allowed the participants to rehearse the spatial information relevant to the subsequent phase and was consistent with our cover story that the experiment was about orientation in the VE. In case they made mistakes, they were shown the correct position.

#### Third Session: Help Request

This phase was the experimental one. The task was to reach a specific spot in the VE, read aloud the plate’s content (visible during this session only), and return to the top of the hill. Depending on the condition, the plate was either inside the building or in the garden. This task was consistent with our cover story that the study’s focus was the spatial navigation and orientation in the VE, but its purpose was to ensure that the participants were in the correct location when the request for help was issued. As the participant moved away from the plate heading back to the hilltop, the request for help started (“Help, help! I am Luca, I am stuck in the cafeteria! Come, come to help me!”); in the fire condition, the request was also preceded by the bursting of the fire. The participant would either decide to ignore the request for help and reach the hilltop as initially instructed, or to comply with the request for help and head to the cafeteria. In the last case, the participant would find a first rescuer in the cafeteria, saying: “I will take over, you can go.” In this way, all participants eventually reached the endpoint, i.e., the top of the hill, and the session closed automatically. We set no time limit to complete the task to avoid that time pressure affected the help rates (Darley and Batson, 1973; Gamberini et al., 2015). To check that the protocol had been followed and the participants had all critical information, we asked about the plate’s content, the victim’s location in the building, and the route to the hilltop. All participants answered correctly.

### Ethics

This study conforms with the Declaration of Helsinki (2013)^4^ and the EU 2016/679 regulation “GDPR.” The participants’ informed consent was collected twice in the study. The participants were initially told that we were studying navigation and orientation in VR. When the data collection for this study was finished, they were debriefed *via* e-mail about the study goal (e.g., studying helping behavior) and could renew their consent by replying “I authorize.” Otherwise, their data were deleted. The data from eight participants were deleted for this reason. The participants underwent no penalty for withdrawing; they were not in a condition of dependability from the researchers conducting the study, nor enrolled in the School in which the researchers teach.

The risks involved in the procedure consisted of experiencing some cybersickness; however, this risk was low since the participants did not have to wear any viewer. We kept the room temperature fresh and advised the participants to interrupt the session if they felt any physical unease. The researcher would offer them some water and keep them company until they could leave. Participants were covered by insurance in case of an accident during the experiment.

Regarding data protection, all data collected was stored in an anonymous format in password-protected hard disks and could only be accessed by the research team. No identification data was kept once the collection period for the second informed consent ended.

### Participants

The data collection took place from March to June 2019. The participants were recruited at the university campus, using the following inclusion criteria: being an Italian native speaker to understand the humanoid’s speech clearly; being Caucasian like the humanoid to prevent any ethnic difference from affecting the help rate (e.g., Dasgupta, 2004); and not having attended any psychology class to be unfamiliar with studies on helping behavior. From the original sample, some participants had to be excluded: three turned out not to meet the inclusion criteria, three did not follow some of the instructions during the experiment, five felt sick during the session, and eight did not reply to the second request for informed consent. The final sample consisted of 62 participants aged 19 to 32 years (*M* = 21.20 SD = 2.39), 14 to 17 participants per condition. This size is in line with the other studies on helping behavior, where an average of 19 participants per condition are involved (Darley and Latanè, 1968; Darley and Batson, 1973; Darley et al., 1973; Clark and Word, 1974; Gaertner, 1975; Gaertner and Dovidio, 1977; Senneker and Hendrick, 1983; Harari et al., 1985; Shotland and Heinold, 1985; Levine et al., 2005; Gillath et al., 2008; Kunstman and Plant, 2008; van den Bos et al., 2009; Slater et al., 2013; Zanon et al., 2014; Gamberini et al., 2015).

Participants were randomly assigned to the experimental conditions. Because of the random assignment and because of the *post hoc* exclusions described above, gender was unevenly distributed across conditions (indoor without fire: women = 5, men = 9; outdoor without fire: women = 8, men = 8; indoor with fire: women = 5, men = 10; outdoor with fire: women = 5, men = 12). The effect of gender will then be assessed in the analysis.

### Data

The VE generated a videoclip animating the sequence of the participants’ Cartesian position on the x, y, and z-axis of the virtual space, sampled every 0.02 s. This video allowed us to determine the occurrence of helping behavior and to double-check that the participants followed the instructions.

The rest of the data was collected *via* e-forms at the beginning and end of the experiment. This self-reported data included: the participant’s gender, age, ethnicity, nationality, native language, attendance of any psychological courses, usage frequency of videogames (1 = Never, 6 = Every day, subsequently re-coded for the analysis into three scores: non-players, infrequent players and players), and pre-session anxiety level (20 items of the state section of the State-Trait Anxiety Inventory, STAI; Spielberger, 2010). We also recorded which researcher run the session.

All statistical analyses were conducted with R-Studio (v. 1.1.463).

## Results

To examine the effect of the two main variables, we used a series of Pearson’s chi-square tests because the number of events per variable available in our study was not adequate for logistic regressions (van der Ploeg et al., 2014).

**FIGURE 2:**
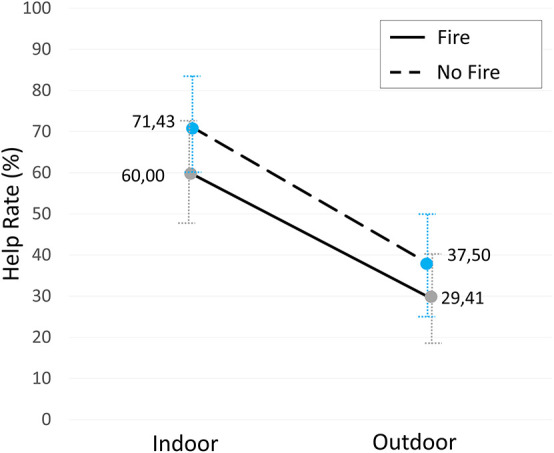
Help rates. Percentage of participants deciding to help (*N* = 62). The blue and gray error bars represent the standard errors.

The help rates observed in the four experimental conditions of our study are displayed in Figure 2. An inspection of the figure suggests that the participants’ location during the help request consistently affected the help rates, which doubled when the participants were indoor. To test the statistical significance of these differences, we conducted a Pearson’s chi-square test comparing indoor (*N* = 32) and outdoor (*N* = 30) conditions; the test returned a statistical significance, χ*2*(1, *N* = 62) = 6.40, *p* = 0.01, confirmed after applying the Yates’s continuity correction, χ*2*(1, *N* = 62) = 5.18, *p* = 0.02. The effect size for this finding was moderate, φ = 0.32.

We then checked the effect of space separately from the effect of the type of emergency. We compared the conditions with fire, indoor (*N* = 15) and outdoor (*N* = 17); separately, we compared the conditions without fire, indoor (*N* = 14) and outdoor (*N* = 16). In both cases, the Pearson’s chi-square test returned a result close to significance, χ*^2^*(1, *N* = 32) = 3.03, *p* = 0.08, with a moderate effect size (φ = 0.31), and χ*^2^*(1, *N* = 30) = 3.45, *p* = 0.06, with a moderate effect size (φ = 0.34). The size of the sample of these two subsets might account for the failure in reaching full significance.

Regarding the effect of the type of emergency on help rates, the inspection of Figure 2 seems to suggest that the presence of fire reduced the help rate by a similar amount in all space conditions. However, the Pearson’s chi-square test returned no statistically significant difference in help rates between fire (*N* = 32) and no-fire (*N* = 30) conditions, χ*2*(1, *N* = 62) = 0.25, *p* = 0.62 (φ = −0.10). We also tested the effect of fire separately from the effect of space. We performed a Pearson’s chi-square test on the outdoor conditions with (*N* = 17) and without fire (*N* = 16), but the difference was not statistically significant, χ2(1, *N* = 33) = 0.24, *p* = 0.62 (φ = −0.09). Likewise, we compared the indoor conditions with (*N* = 15) and without fire (*N* = 16) using Fisher’s exact test because the expected frequency for the “no help” outcome in the no-fire group was less than 5 (Fe = 4.85). Again, the effect of fire was not statistically significant (*p* = 0.70, *φ* = −0.12).

A few controls were then run. We considered the effect on help rates of three variables, i.e., the participant’s gender (female or male), and expertise (non-players; infrequent players; frequent players), and the experimenter running the session (A or B). None showed a relationship with the help rates (Table 1), and the effect size tested with Cramer’s φ for gender and experimenter and Cramer’s V for expertise (*df* = 2) was small (*φ* < 0.30; *V* < 0.21).

**TABLE 1:** Results of the Pearson’s chi-square tests (control variables).

Variable	Level	*N*	Help frequency (%)	χ2	Df	*p*	Effect size*
Gender	Female	23	47.83%	0.01	1	0.95	φ = 0.01
	Male	39	48.72%				
Expertise	Non-Players	28	42.86%	1.73	2	0.42	V = 0.17
	Infrequent Players	16	62.50%				
	Players	18	44.44%				
Experimenter	A	31	45.16%	0.26	1	0.61	φ = −0.07
	B	31	51.61%				

*Expertise has more than 2 levels, so Cramer’s V was used for calculating its effect size.

**N*, number of participants in the sample belonging to the variable level.*

*Help frequency (%) = percentage frequency of participants in the variable level deciding to help.*

Finally, we considered the anxiety scores across the four conditions: indoor with fire (*N* = 15, mean rank = 23.20), indoor without fire (*N* = 14, mean rank = 31.79), outdoor with fire (*N* = 17, mean rank = 34.12), and outdoor without fire (*N* = 16, mean rank = 36.25). A Shapiro–Wilk test revealed that the anxiety scores’ distribution was not normal in one condition (indoor without fire), *W* = 0.77, *p* = 0.002, violating the ANOVA normality assumption. Thus, we used a Kruskal–Wallis test to compare the anxiety scores between conditions; no significant differences were found, *H*(3) = 4.68, *p* = 0.20 (η*^2^* = 0.03). The *post hoc* test, performed with the “kruskalmc” function of the “pgirmess” package as proposed by Siegel and Castellan (1988), confirmed this result also with pairwise comparisons (Table 2). None of the observed differences was greater than the critical difference, and the effect size associated with the comparisons was small (Vargha and Delaney’s *A* < 0.56). Therefore, the possibility that the groups of participants assigned to the different conditions differed in their level of anxiety was discarded.

**TABLE 2:** *Post hoc* test on the STAI scores.

Comparison	Observed difference*	Critical difference**	*A* ***
Indoor—fire vs. Indoor—no fire	8.59	17.69	0.36
Indoor—fire vs. Outdoor—fire	10.92	16.86	0.31
Indoor—fire vs. Outdoor—no fire	13.05	17.11	0.31
Indoor—no fire vs. Outdoor—fire	2.33	17.18	0.47
Indoor—no fire vs. Outdoor—no fire	4.46	17.42	0.42
Outdoor—fire vs. Outdoor—no fire	2.13	16.58	0.46

* the difference observed between the mean ranks of the conditions included in the comparison.

**the difference that would be associated with statistical significance at *p* < 0.05

***the threshold for medium effect is.56.

In conclusion, our hypothesis that sharing the space with the victim increased the help rate was confirmed on the whole sample and seemed unaffected by the type of emergency.

## Discussion

According to Latanè and Darley (1970), interpreting the situation as an emergency is necessary for providing help. Thus, a possible explanation for our results is that, in the indoor conditions, the participants *recognized* the emergency more easily. However, the help request and, when present, the fire effects were equally perceivable from any position, indoor or outdoor. Another possible explanation of our results could be that the route to the victim looked more difficult in the outdoor conditions, thereby increasing the perceived *cost* of providing help (Piliavin et al., 1981). However, we have no evidence that dexterity affected the participants’ decision to help. For instance, the participants’ expertise with games did not affect their decision to help. Also, they could practice the needed route in the previous phase of the experiment.

We think that the explanation that better fits our study is that the different spatial arrangements worked as a social categorization device; in other words, being inside the same building created a common ingroup identity for potential helper and victim (Gaertner et al., 1993; Dovidio et al., 2010), making the former more likely to act pro-socially. The effect of belonging to the same spatial formation on social identity is not explored directly in our study but is consistent with its findings and the tenets of social categorization theory, as explained in the introduction. The role of spatial boundaries in identifying an ingroup could also account for other reported phenomena, such as the sense of belonging to an environment and the related loyalty and affection to it (Kolesovs, 2021) or the pro-social behavior in people synchronizing their movements in space and time to a common rhythm (Cross et al., 2019).

In conclusion, our results have a methodological import, suggesting to control the victim and the helper’s mutual positions in studies of helping behavior. Moreover, they suggest that social identity can be implied in the effect of space on pro-social behavior, and this explanation is worth further investigation. Finally, by highlighting the role of space in pro-social behavior, our study has added one more reason why VR can be helpful to this line of research. VR allows to manipulate space easily, as testified by other lines of the psychology of space that have already adopted VR in their toolkit (e.g., spatial behavior, Durlach et al., 2000; spatial abilities, Dünser et al., 2006).

## Data Availability Statement

The original contributions presented in the study are included in the article/Supplementary Material, further inquiries can be directed to the corresponding author/s.

## Ethics Statement

Ethical review and approval was not required for the study on human participants in accordance with the local legislation and institutional requirements. The patients/participants provided their written informed consent to participate in this study.

## Author Contributions

AS and LG conceived and planned the work and the methodological approach. MMr developed the virtual environment. MF and MMs collected the data. MMs and PP analyzed the data. AS, LG, MF, and MMs wrote the manuscript. All authors contributed to the article and approved the submitted version.

## Conflict of Interest

The authors declare that the research was conducted in the absence of any commercial or financial relationships that could be construed as a potential conflict of interest.

## Publisher’s Note

All claims expressed in this article are solely those of the authors and do not necessarily represent those of their affiliated organizations, or those of the publisher, the editors and the reviewers. Any product that may be evaluated in this article, or claim that may be made by its manufacturer, is not guaranteed or endorsed by the publisher.

## Footnotes

1
https://www.flaimsystems.com

2
https://www.onr.navy.mil/en/Media-Center/Press-Releases/2018/ONR-TechSolutions-Flight-Deck-Crew-Refresher- Training

3
https://www.unrealengine.com

4
https://www.wma.net/policies-post/wma-declaration-of-helsinki-ethical-principles-for-medical-research-involving-human-subjects/
