# Supplementary figures and images for: Sharing the Space With the “Victim” Can Increase Help Rates. A Study With Virtual Reality
Source: Front Psychol. 2021 Sep 8;12:729077. doi: 10.3389/fpsyg.2021.729077 (PMC8455842; doi:10.3389/fpsyg.2021.729077)

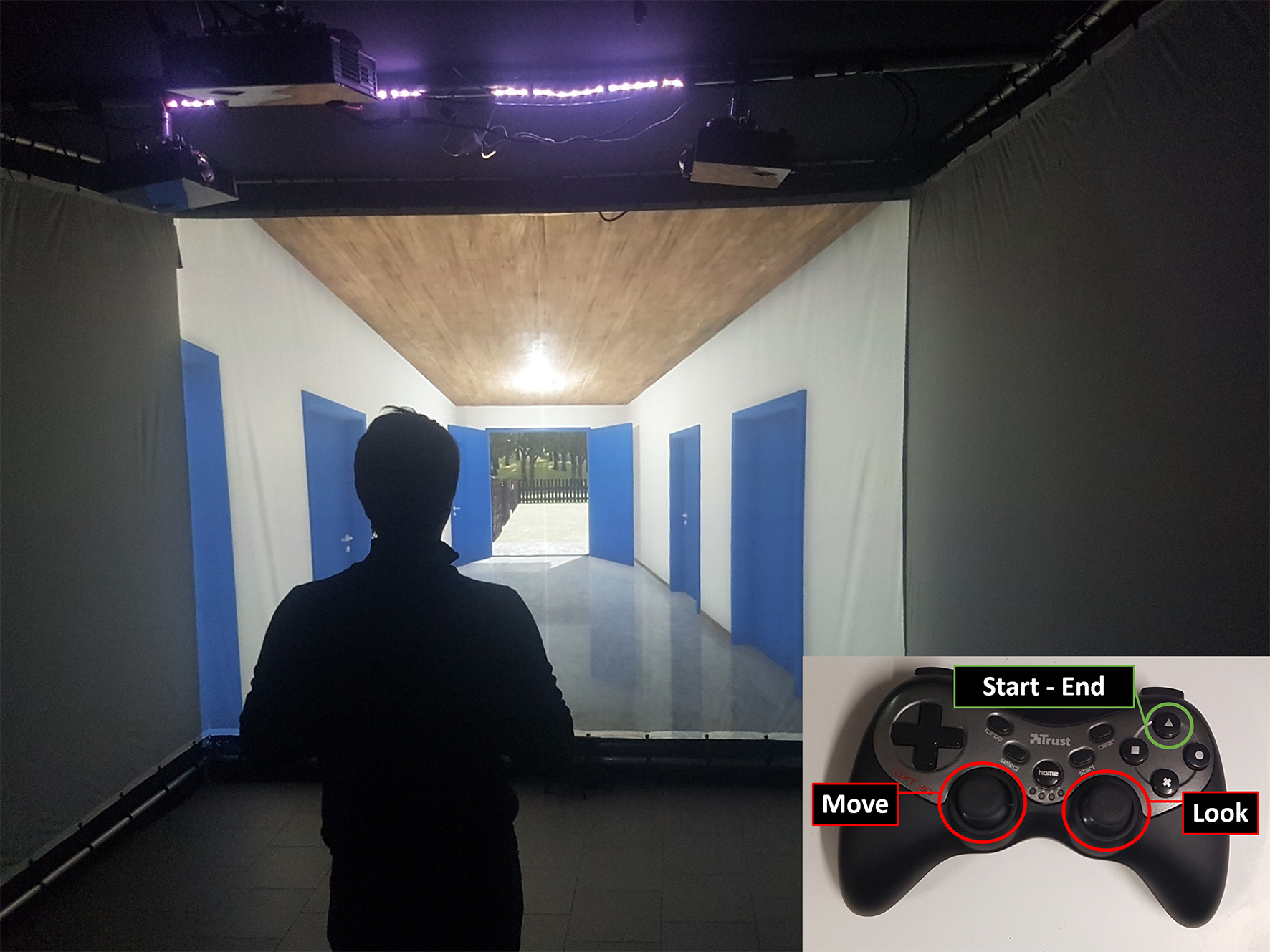

Supplement: Supplementary file 1 [file Image_1.TIF]
